# RESTseq – Efficient Benchtop Population Genomics with RESTriction Fragment SEQuencing

**DOI:** 10.1371/journal.pone.0063960

**Published:** 2013-05-17

**Authors:** Eckart Stolle, Robin F. A. Moritz

**Affiliations:** 1 Institute of Biology, Department of Zoology, Martin-Luther-University Halle-Wittenberg, Halle (Saale), Germany; 2 Bio-Solutions GmbH, Martin-Luther-University Halle-Wittenberg, Halle (Saale), Germany; 3 Department of Zoology and Entomology, University of Pretoria, Pretoria, South Africa; 4 Facultatea de Zootehnie şi Biotehnologii, Universitatea de Ştiinţe Agricole şi Medicină Veterinară, Cluj-Napoca, Romania; University of Sydney, Australia

## Abstract

We present RESTseq, an improved approach for a cost efficient, highly flexible and repeatable enrichment of DNA fragments from digested genomic DNA using Next Generation Sequencing platforms including small scale Personal Genome sequencers. Easy adjustments make it suitable for a wide range of studies requiring SNP detection or SNP genotyping from fine-scale linkage mapping to population genomics and population genetics also in non-model organisms. We demonstrate the validity of our approach by comparing two honeybee and several stingless bee samples.

## Introduction

The development of new techniques for Next Generation Sequencing (NGS) platforms has revolutionized marker assisted studies in the past few years. Its use for SNP genotyping opened the way for high resolution genomic studies on a population level to investigate evolutionary processes and genotype-phenotype relationships. Genotyping, by sequencing fractions of a genome, has become widespread and allowed for recent ecological, evolutionary, phylogeographic and genetic mapping studies based on both large numbers of SNPs and large numbers of individual samples [1]. Nevertheless, partial or complete sequencing of large genomes of non-model organisms remains a costly enterprise. For many questions it is sufficient to saturate the genome with SNP markers at sufficiently high density and various techniques using restriction endonucleases have been proposed to reduce the sequencing efforts and the complexity of DNA libraries. Restriction-site associated DNA, RAD [2], with its derivatives 2b-RAD [3] and double digest RADseq [4], or genotyping by sequencing, GBS [5], reduced representation libraries, RRL [6], complexity reduction of polymorphic sequences, CRoPS [7] and multiplexed shotgun genotyping, MSG [8] were used in a number of studies[1], [9]–[11]. All employ a digestion of the DNA sample with various subsequent preparation steps. However, they require the use of high throughput next generation sequencing platforms allowing for high read numbers. With the development of smaller platforms (typically below 10 million reads per run [12]), benchtop machines are now available to be operated also in smaller scale genetic and ecological laboratories. Unfortunately, most of the established techniques cannot efficiently be implemented for high quality and/or high density SNP marker genotyping in multiple samples due to an insufficient number of reads. Moreover, there are other limitations in the established marker enrichment procedures including the amount of DNA needed, cumbersome library preparation, bias introduced by PCR steps or the reproducibility of the library preparation. The choice of rare cutting restriction enzyme(s) may result in a biased distribution of the sequenced DNA fragments, causing larger gaps between markers across the genome.

Here we present a novel approach termed restriction fragment sequencing (RESTseq) to overcome the shortcomings of the existing methods. It allows for marker enriched SNP typing on small scale platforms and can increase the efficiency for analyzing large numbers of barcoded samples in high-throughput systems. Our approach leads to an efficient reduction of the complexity of the prepared libraries. The method yields fragments potentially containing SNPs with an unbiased distribution across the genome. As a result SNP genotypes are obtained at much less sequencing effort reducing costs for chemicals and labor. The method is especially suitable for population genomic studies on small scale sequencing platforms like Ion Torrent PGM (Life Technologies), MiSeq (Illumina) and 454 GS Junior (Roche). Alternatively, sequencing depth or level of pooling of barcoded samples can be increased on large scale platforms like Illumina HiSeq, ABI Solid or Roche 454 GS FLX.

## Methods

### DNA Sources

We randomly sampled two worker (diploid females) individuals from a single honeybee (*Apis mellifera*) colony in Halle (Germany) for the validation of RESTseq. Four stingless bee males of *Nannotrigona perilampoides* were sampled from a drone congregation in Mérida (Mexico). Whereas the honeybee samples were freshly sampled, the stingless bees were sampled and stored in ethanol (100%) for about 6 month at room temperature. Genomic DNA was extracted using a standard solvent extraction with Phenol-Chloroform [13].

### Library Preparation and Sequencing

Up to two micrograms genomic DNA were digested with *Taq*I in a 30 µl reaction. The reaction was incubated at 65°C with 100 U *Taq*I for 5 hours to ensure complete restriction. After digestion, the reaction was purified with AMPure (Agencourt) and processed according to the manufacturer’s instructions for the IonTorrent platform (Ion Plus Fragment Library Kit). The library then was digested with 100 U *Tru*I (*Mse*I isoschizomere) at 65°C for 5 hours. The reaction then was directly electrophoretically size selected with a Pippin Prep (Sage Science, setting: 170 bp “tight”, resulting in a selected range of about 70 bp –105 bp for the target excl. adapter) using a 2% ethidiumbromide-free cartridge. The recovered fragments were quantified by real-time PCR (Ion Torrent library quantification kit), diluted, and used for sequencing according to the manufacturer’s instructions. A final PCR amplification of the library was not necessary, because we had a sufficient yield of DNA.

From each of the *N. perilampoides* samples, 50 ng DNA was digested in a total volume of 25 µl with 50 U *Taq*I and 5 U RNaseH at 65°C for 2 hours. Ligation and nick repair was performed by adding 50 U T4 Ligase (Invitrogen), 0.1 µl ATP (100 mM), 0.5 T4 Ligase Buffer (10x), 0.3 µl (30 µM) of IonTorrent P1 and 0.3 µl (30 nM) IonTorrent A adapter, both with sticky ends compatible to the *Taq*I overhang. The latter contained a barcode before the sticky end (barcodes X1, X3, GBE2, GBE4, see table S1). After 45 min incubation at room temperature, 2 µl dNTPs (10 mM), 25 U *Taq* polymerase (PeqLab), 10 µl *Taq* polymerase buffer (10x) and 33 µl water were added and incubated at 70°C for 10 min. After pooling all reactions and a purification with AMpure, we added to 30 µl purified sample 50 U *Mse*I, 10 U of each *Mlu*CI, *Hae*III, *Msp*I and *Hin*P1I (all NEB), 4 µl NEB4 Buffer (10x), 0.4 µl BSA (100x) and 1.1 µ water. Six hours incubation at 37°C was followed by AMpure purification, PCR (IonTorrent Xpress Fragment library kit, High Fidelity PCR mix and primer, 15 cycles) and size selection (265 bp “tight”, Pippin Prep) before quantification and sequencing.

### Data Analysis

Sequence reads were automatically quality filtered (cutoff 15) by the Ion Torrent analysis pipeline (Torrent Suite version 3.2.1). The resulting high quality reads were further analyzed with the CLC Genomics Workbench software (version 5.5.1, CLC Bio) and reads not starting with CGA (*Taq*I recognition site) were discarded. We then performed a reference sequence based analysis where we mapped reads to the *Apis mellifera* 4.5 assembly (234 Mb) with the following settings: mismatch cost 3, deletion cost 3, insertion cost 3, length fraction 0.95, similarity fraction 0.95, non-global, unspecific reads ignored. A subsequent quality-based variant detection was performed with the settings neighborhood radius 5, maximum gap and mismatch count 2, minimum neighborhood quality 15, minimum central quality 20, minimum coverage 10, minimum variant frequency 0.4, requirement of presence in both forward and reverse reads (at least 5% of the occurrences of the detected variant) and with a filter for 454/Ion Torrent homopolymer indels.

The *N. perilampoides* high quality reads were debarcoded and the reads were filtered for those containing the *Taq*I recognition site on both ends. Since the barcodes were 4 or 10 bp long, but the pooled library was size selected simultaneously, we selected only reads between 163 and 207 bp (selected size 240–290 bp minus 77 or 83 bp for the barcoded adapters). *De novo* assembly was performed with CLC Genomics Workbench (version 6.0.2, word size 30, minimal contig size 160 bp) and mapping of the reads from each barcode was performed with the following settings: mismatch cost 2, deletion cost 2, insertion cost 2, length fraction 0.9, similarity fraction 0.9, non-global, unspecific reads ignored.

Sequences were deposited in the NIH Short Read Archive (SRA accession numbers SRP020236 and SRP020478).

## Results and Discussion

**Figure 1:**
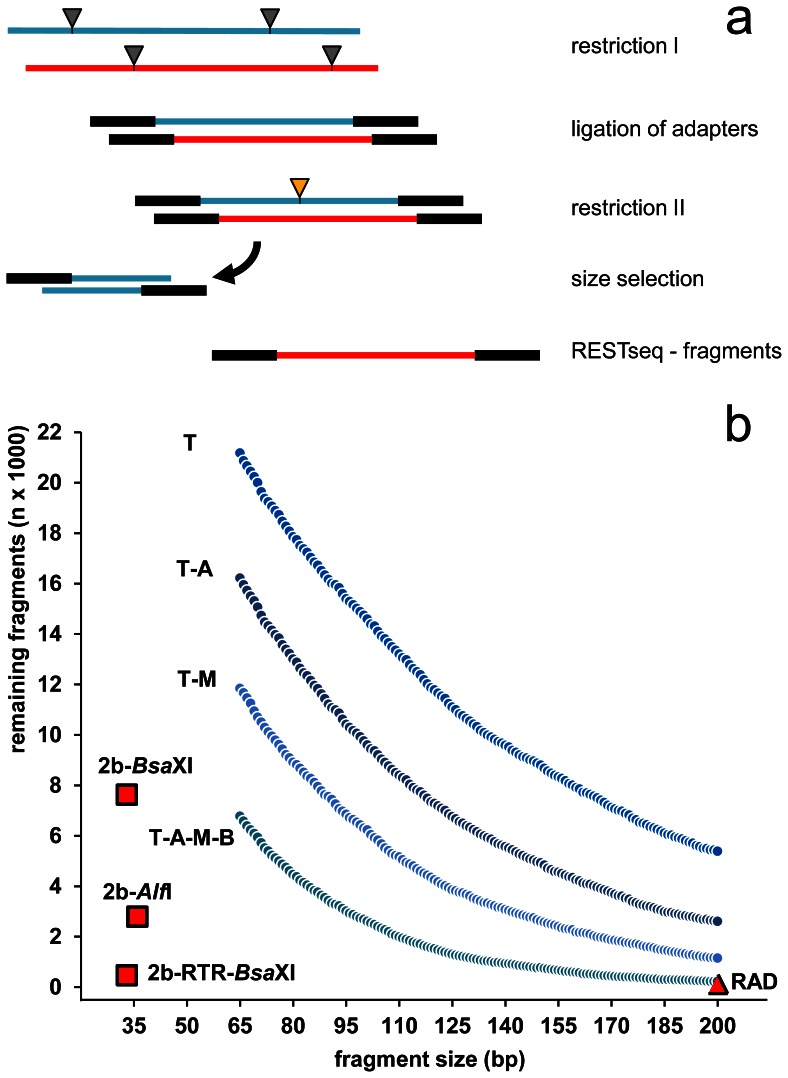
Principle of RESTseq library preparation and the relationship between selected fragment size, chosen restriction endonucleases, and remaining fragment number. (**a**) Library preparation is accomplished by restriction I, ligation of sequencing adapters, restriction II and then size selection. (**b**) Illustration of the possible reduction of fragment number and thus library complexity by selecting either longer fragments or by the choice of restriction endonuclease combinations. Shown are expected fragment numbers from *A. mellifera* LG1 (29.9 Mbp) when choosing fragment size ranges of 20 bp (+/−10 bp around the selected size) and using *Taq*I (T, TˆCGA) for restriction I in combination with either *Apo*I (A, RˆATTY) or *Mse*I (M, TˆTAA) for restriction II or the combination *Apo*I/*Mse*I/*Bst*UI (B, CGˆCG). Numbers of the 33 bp and 36 bp long *Bsa*XI and *Alf*I fragments (2b-RAD [3]) are shown for comparative reasons as well as the number of *Sbf*I fragments (commonly used for RAD sequencing [2], [15]).

The method starts with the digestion of genomic DNA with a frequent cutting restriction endonuclease to generate a high number of fragments (Fig. 1a). Following the ligation of platform-specific sequencing adapters, a second digestion with another frequent cutting restriction endonuclease is further reducing the library in complexity. The only obligate constraint is that this enzyme may not have a restriction site within the ligated sequencing adapters. This cleaves the majority of the previously generated fragments. The remaining uncleaved fragments are recovered in a size selection step, removing cleaved fragments and adapter dimers from the sample. If necessary, an additional PCR can be used to amplify the library using the suitable primers matching adapter sequences on either end. The remaining uncleaved fragments are favored over potential traces of cleaved fragments due to exponential versus linear amplification. The retrieved library can be sequenced according to the manufacturer’s instructions.

**Figure 2:**
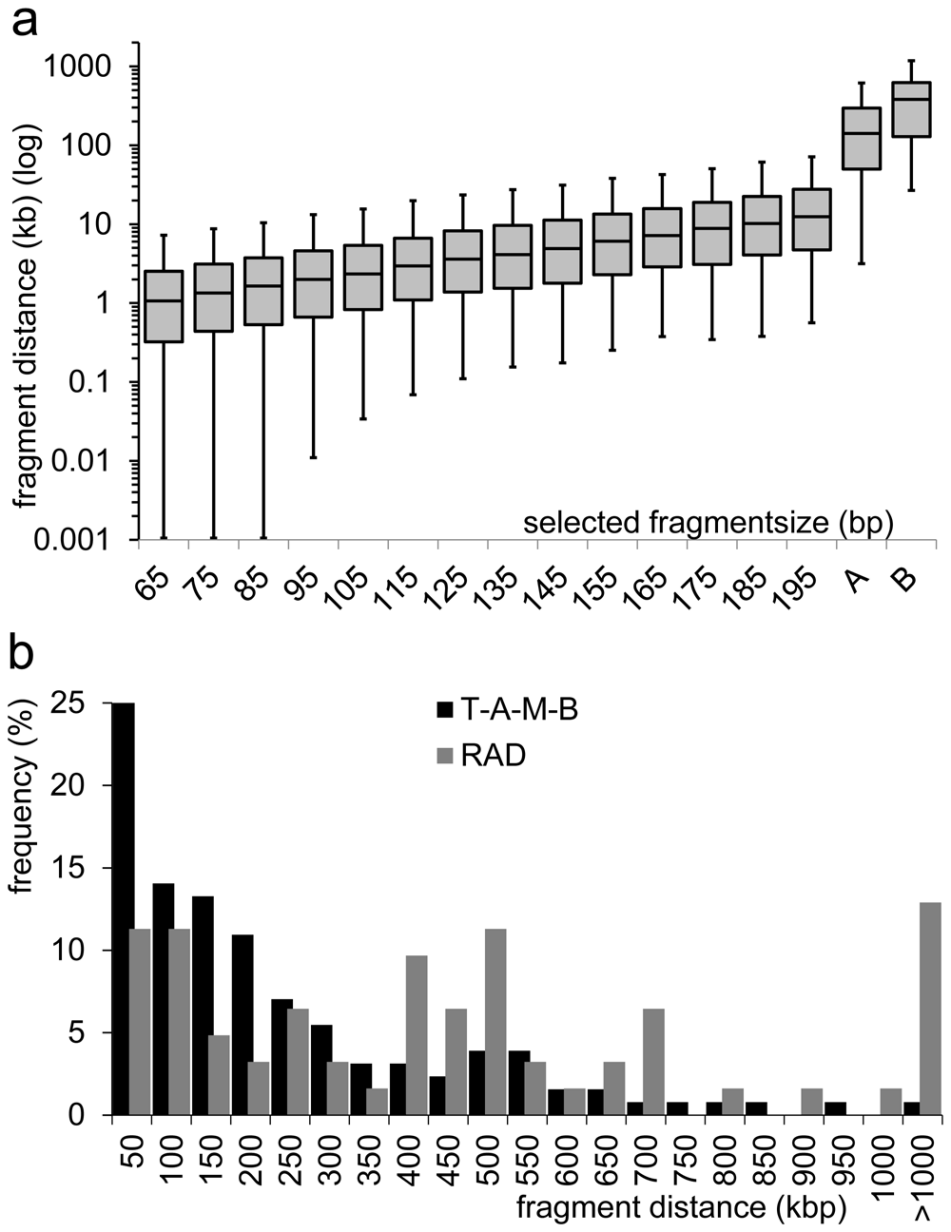
Distances of RESTseq fragments. (**a**) Illustration of interfragment distances in relation to the selected fragment size (range of 20 bp) and thus numbers of the fragments (cf. Fig. 1b). The Box-Whiskers-Plot shows the median with 25^th^–75^th^ percentile and 5–95% as errorbars. For comparative reasons we further included (A) 129 fragments obtained from a *Taq*I library which was reduced by *Mse*I, *Apo*I and *Bst*UI and selected for 190–200 bp and (B) 124 fragments obtained from 62 *Sbf*I recognition sites on *A. mellifera* LG1. (**b**) Frequencies of distances between 129 fragments obtained from a *Taq*I library which was reduced by *Mse*I, *Apo*I and *Bst*UI (T-A-M-B) and selected for 190–200 bp and 124 fragments obtained from 62 *Sbf*I recognition sites (RAD) on *A. mellifera* LG1.

This combination of the chosen restriction endonucleases and the range for the size selection not only reduces the complexity of a DNA sample most efficiently but also allows for a most flexible adaptation matching the specific requirements of the experiments and the sequencing platforms in terms of number and length of the fragments (Fig. 1b,2a). For organisms with sufficient genome sequence data available, the choice of suitable restriction enzymes can easily be tested *in silico* for the desired number of SNPs to be detected per individual sample and/or across a population. If less SNP markers are required, the size selection can be modified and/or aim at larger fragments which are less frequent. It is also possible to use additional restriction endonucleases in the second digestion step, which further reduces the number of fragments (Fig. 1b). Even under extremely strong reduction, fragments are still randomly distributed within the genome whereas those obtained from rare cutting endonucleases show non-random distribution with large gaps (Fig. 2a,b). RESTseq provides Poisson distributed fragments, whereas the *Sbf*I restriction fragments significantly deviate from a random distribution. The setup is thus very flexible and can be fine-tuned to the very specific questions and/or sequencing conditions/requirements.

We verify the RESTseq method by showing that we can reproduce a majority of sequenced fragments and also SNPs with an identically prepared biological replicate. For the Ion Torrent PGM platform we prepared two honeybee libraries using *Taq*I (TˆCGA) and *Mse*I (TˆTAA) for the first and second restriction, respectively, together with a size selection around 90 bp. The unamplified libraries were sequenced each on a 316 chip and generated 3.67 and 2.71 million reads of 83 and 86 bp average length, respectively. 99% of the reads started with the correct triplet CGA from the *Taq*I recognition site. The use of *Mse*I resulted in an enrichment of GC-rich fragments by reducing AT-rich fragments due to cleavage. Whereas the overall GC content of the *Apis mellifera* genome is 32%, both libraries had a higher GC content of 44% which might enrich for fragments from coding regions, highly desirable when screening populations for patterns of selection. With conservative settings, 72% and 77% of the reads (average length 85.2 and 89.5 bp) mapped unambiguously to the *A. mellifera* genome and covered 11.06 and 10.05 Mbp at a 20x and 17x coverage, respectively. The mean genome-wide distance between mapped fragments was 1.8 and 2.0 kbp (max. 138.7 and 146.2 kbp), respectively. Given that the honeybee genome still contains various large assembly gaps of 50 kb between genomic scaffolds, the real distance between the fragments might be even smaller. A conservative quality-based variant detection detected in total 24755 and 19191 SNPs (>90%), InDels or multi-nucleotide variants, respectively. For the first sample the variants had a mean distance of 7.75 kbp (median: 2.3 kbp, max.: 234 kbp) which, with the high recombination rate of 15.7 cM/Mb in *A. mellifera* [14], would relate to a mean genetic distance of 0.12 cM (max 3.67 cM) – by far sufficient to allow for high resolution genomic studies.

**Figure 3:**
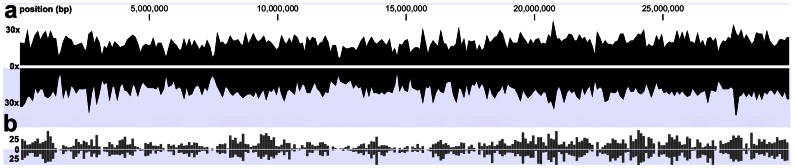
Reproducibility of RESTseq by comparison of two libraries with an IonTorrent. (**a**) read coverage after mapping both sequenced libraries runs on *A. mellifera* LG1. (**b**) Respective variant detection for both libraries.

We compared the quality of both sequencing runs for linkage group 1 of the *A. mellifera* genome (29.89 Mbp, GC content 31.2%, Fig. 3a,b). Our aim was to sequence the fragments of 80–100 bp length (expected n = 7431, cumulative length 707.5 kbp, GC content 43.3%, located in genes 423.6 kbp, located in CDS regions 32 kbp) in both samples in order to genotype the same genomic regions. Mapping the obtained reads against this set of fragments as reference, yielded 227 K and 211 K mapped reads (mean length 88 bp) covering 626.6 kpb (89%) and 639.8 kbp (90%) of the target, respectively. Both combined covered 93% of the target, whereby the overlap between both samples was 603.2 kbp (85.3% of the target) proving a high reproducibility. With read coverage of 32x and 29x, 2195 and 2323, respectively, high quality variants were detected. If we perform a *de novo* assembly from the combined reads from both runs and map the resulting contigs against the target reference, with 5645 contigs (mean length 89.6 bp) we reach a consensus of 501.81 kb and hence 71% of the reference. This confirms that a *de novo* approach as such, is feasible to generate sufficient quality data for sound analyses, albeit yielding less consensus sequence than a reference based approach due to the assembly computing. Nevertheless, RESTseq is clearly also feasible for non-model organisms without a sequenced reference genome. Based on our empirical results for the detection of the target region, we calculated a sequencing effort of 51.4 kpb and 35.8 kbp to cover 1 kbp target which is almost 5 times lower than what we calculate for other techniques like 2b-RAD [3] or GBS [5], [10] which require about 150 kbp sequence per 1 kbp target.

Although our approach is similar to GBS [5] or double digest RAD [4], it has the advantage of a direct control over the complexity of the library due to the digestion after adapter ligation as well as the more unbiased fragment distribution. However, certain aspects of the already described approaches can easily be adapted. Population genetic studies, for example, require often a limited number of markers combined with high levels of barcoding in order to genotype large numbers of individuals. Following our validation example and *in silico* calculations, a reduction of an *Apis mellifera Taq*I library with a set of five restriction endonucleases (*Mse*I (TTAA), *MluC*I (AATT), *Bsh*1236I (CGCG), *Msp*I (CCGG), *Hin*P1I (GCGC), identical buffer and temperature requirements allows parallel usage) in combination with a size selection would leave about 155–195 bp yields about 2250 fragments covering almost 400 kb of the genome. These fragments can be covered by a single read as the platform offers read-lengths of 200 bp and more and the amount of sequence should be sufficient to provide polymorphism data for a couple of hundred loci. Using barcoded adapters (e.g. GBS or IonTorrent barcoded with exception of those harboring a restriction site, Table S1), about 60–80 of such libraries could be pooled to be genotyped with a single IonTorrent run such as described above. Hence this approach opens up possibilities for large scale population genetic studies and is thus capable to replace the usage of microsatellite genotyping. Such a set-up was tested on four males of *Nannotrigona perilampoides*, a stingless bee for which no genomic information is available. We generated the initial *Taq*I libraries with barcoded adapters, reduced the pooled library with a combination of five enzymes (*Mse*I, *Mlu*CI, *Hae*III, *Msp*I, *Hin*P1I) and performed a size selection around 265 bp after PCR. A sequencing run on a 316 IonTorrent chip yielded 3.2 million barcoded reads (1.06, 0.41, 0.26 and 1.47 million reads for each barcode, respectively) of which a subset was used for a *de novo* assembly. Using the resulting 4379 contigs (L = 792kbp) as read mapping reference, large fraction could be covered at high read coverage (78% at 179x, 68% at 70x, 73% at 46x, and 90% at 209x) and 726 SNPs (min. frequency 20%) could be detected. The barcodes in this experiment were 10, 10, 4 and 4 bp long, respectively, thus it is likely that significant parts of the variation in covering the target region are owed to the pooling of the libraries prior to size selection. Although the use of barcodes of identical length, the application of better suited *de novo* assembly and mapping algorithms might improve the yield, our results show that even with low read numbers a target region of less than 1 Mbp can be covered efficiently (0.26 million reads covering 73% of 0.8 kbp at 46x read coverage). For ddRAD [4] it was reported that more than 0.2 million reads sufficed to cover a shared region of 14–17 kbp. Thus, the presented approach of using RESTseq with barcoded samples and strong, but efficient library complexity reduction by combining several restriction endonucleases, is well suited for population genetic analysis of multiple samples in non-model organisms. The high degree of complexity reduction of libraries and reproducibility makes this method particularly suitable for using benchtop sequencing machines. The comparatively low capacities of such machines can be used more efficiently with our method, providing the possibility to run sufficient numbers of samples in short time and at low cost. However, with some adaptations to the platform, the presented method can readily be used for larger sample numbers on high throughput machines as well.

### Conclusion

All in all RESTseq provides a simple (and therefore robust), yet highly flexible tool that allows genome wide analyses also on low budget benchtop sequencers. The advantages over alternative methods are the lower sequencing effort, the unbiased distribution of fragments across the genome and the high flexibility to be adapted to specific questions and platform capacities. It is scalable for either population genomics or linkage association in mapping studies both in model and non-model organisms without a reference genome available. RESTseq has the potential to become a standard tool in population genetics and molecular ecology replacing microsatellite DNA analyses as these replaced allozyme marker studies in the 1980s.

## Supporting Information

Table S1 Barcodes without recognition sites. The list contains IonTorrent barcode names (Ion Xpress™ Barcode Adapters 1–96 Kit, Life Technologies, sequences are available there) and barcode sequences published for GBS [5], which do not contain or form a recognition site of *Taq*I, *Mse*I, *Mlu*CI, *Hae*III, *Msp*I, *Hin*P1I, *Mbo*I, *Rsa*I or *Bsh*1236I if combined with an IonTorrent adapter at the 5′ end and/or CCGA at the 3′ end (modified *Taq*I TCGA site to not restore the site after ligation).
(XLSX)
